# Weighted quantitative isoperimetric inequalities in the Grushin space ${R}^{h+1}$ with density $|x|^{p}$

**DOI:** 10.1186/s13660-017-1437-5

**Published:** 2017-07-12

**Authors:** Guoqing He, Peibiao Zhao

**Affiliations:** 1 School of Science, Nanjing University of Science and Technology, Nanjing, 210094 China; 2 School of Mathematics and Computer Science, Anhui Normal University, Wuhu, 241000 China

**Keywords:** 52B60, 53C17, Grushin space, density, quantitative isoperimetric inequality

## Abstract

In this paper, we prove weighted quantitative isoperimetric inequalities for the set $E_{\alpha}= \{(x,y)\in {R}^{h+1}: \vert y \vert <\int_{\arcsin \vert x \vert }^{\frac{\pi}{2}}\sin^{\alpha +1}(t)\,dt, \vert x \vert <1 \}$ in half-cylinders in the Grushin space ${R}^{h+1}$ with density $\vert x \vert ^{p}$, $p\geq0$.

## Introduction

The study of isoperimetric problems in Carnot-Carathéodory spaces has been an active field over the past few decades. Pansu [1] first proved an isoperimetric inequality of the type $P_{H}(E)\geq C \vert E \vert ^{\frac{3}{4}}$ ($C>0$) in the Heisenberg group $\mathbb{H}^{1}$ where $P_{H}(E)$ and $\vert E \vert $ denote Heisenberg perimeter and Lebesgue volume of *E*, respectively. In 1983 Pansu [2] conjectured that, up to a null set, a left translation and a dilation, the isoperimetric set in the Heisenberg group $\mathbb{H}^{1}$ is a bubble set as follows:

1 $$ E_{\mathrm{isop}}= \biggl\{ (z,t)\in\mathbb{H}^{1}: \vert t \vert < \frac{1}{2}\bigl(\arccos \vert z \vert + \vert z \vert \sqrt{1- \vert z \vert ^{2}}\bigr), \vert z \vert < 1 \biggr\} . $$

The formula defining $E_{\mathrm{isop}}$ in (1) makes sense in $\mathbb{H}^{n}$ for $n\geq2$ and Pansu’s conjecture can be naturally extended to any dimension. Until today Pansu’s conjecture has not completely been solved. It has been only supported by many partial results, where further hypotheses involving regularity or symmetry of the admissible sets are assumed; see [3–8]. For Carnot groups, one can only get an isoperimetric inequality [9] though we know the fact that isoperimetric sets exist [10].

Monti and Morbidelli [11] completely solved the isoperimetric problem in the Grushin plane ${R}^{2}$. Franceschi and Monti [12] studied isoperimetric problem for a class of *x*-spherical symmetry sets (here if $h=1$, the assumption of *x*-spherical symmetry can be removed) in Grushin spaces ${R}^{h+k}$. In particular, they pointed out that when $k=1$, up to a null set, a vertical translation and a dilation, the *x*-spherical symmetric isoperimetric set is

2 $$ E_{\alpha}= \biggl\{ (x,y)\in{R}^{h+1}: \vert y \vert < \int_{\arcsin \vert x \vert }^{\frac{\pi}{2}}\sin^{\alpha+1}(t)\,dt, \vert x \vert < 1 \biggr\} . $$

In the case of $\alpha=1$, the set $E_{\alpha}$ (2) is just the Pansu sphere in the Heisenberg group.

On the other hand, manifolds with density, a new category in geometry, have been widely studied. They arise naturally in geometry as quotients of Riemannian manifolds, in physics as spaces with different media, in probability as the famous Gauss space and in a number of other places as well (see [13, 14]). Morgan and Pratelli [15] studied the isoperimetric problems in Euclidean spaces ${R}^{n}$ with density; see [16–20] and the references therein. The weighted Sobolev and Poincaré inequalities for Hörmander’s vector fields were well studied in [21–23]. The weighted isoperimetric-type and Sobolev-type inequalities for hypersurfaces in the Carnot group with density were obtained in [24]. In [25] He and Zhao proved that the set $E_{\alpha}$ is also a weighted *x*-spherical symmetry isoperimetric set in the Grushin space ${R}^{h+1}$ with density $\vert x \vert ^{p}$, $p> -h+1$.

Very recently, Franceschi *et al.* [26] obtained quantitative isoperimetric inequalities for the bubble set $E_{\mathrm{isop}}$ in half-cylinders in Heisenberg groups by the construction of sub-calibrations.

Motivated by the nice work mentioned above, in this paper we consider the quantitative isoperimetric inequalities for the set $E_{\alpha}$ in half-cylinders in the Grushin space ${R}^{h+1}$ with density $\vert x \vert ^{p}$, $p\geq0$. These inequalities show that the weighted volume distance of a set *F* from the set $E_{\alpha}$ with the same weighted volume is controlled in terms of the difference of the weighted *α*-perimeter of *F* and the weighted *α*-perimeter of $E_{\alpha}$. We get the following theorem.

### Theorem 1.1

*Let* *F* *be any measurable set in the Grushin space* ${R}^{h+1}$ *with density* $e^{\phi}= \vert x \vert ^{p}$, $p\geq0$, *where* *F* *satisfies* $V_{\phi}(F)=V_{\phi}(E_{\alpha})$. *Let* $C_{\varepsilon}= \{(x,y)\in {R}^{h+1}: \vert x \vert <1, y>y_{\varepsilon}\}$ *be half*-*cylinders*, *where* $0\leq\varepsilon<1$ *and* $y_{\varepsilon}=f(1-\varepsilon)$ *with* $f(r)=\int_{\arcsin r}^{\frac{\pi}{2}}\sin^{\alpha+1}(t)\,dt$.

(i) *If* $F\triangle E_{\alpha}\subset\subset C_{0}$, *then we have*
  $$P_{\alpha,\phi}(F)-P_{\alpha,\phi}(E_{\alpha})\geq\frac{h+p}{120\omega _{h}^{2}}V_{\phi}(E_{\alpha}\triangle F)^{3}. $$
(ii) *If* $F\triangle E_{\alpha}\subset\subset C_{\varepsilon}$ *with* $0<\varepsilon<1$, *then we have*
  $$P_{\alpha,\phi}(F)-P_{\alpha,\phi}(E_{\alpha})\geq\frac{\sqrt{\varepsilon }(h+p)}{8 [(1-\varepsilon)^{\alpha+h}+(1-\varepsilon)^{h}\sqrt {\varepsilon} ]\omega_{h}}V_{\phi}(E_{\alpha}\triangle F)^{2}. $$
  *Here* $P_{\alpha,\phi}(E)=\sup \{\int_{E} \operatorname{div}_{\alpha}( \vert x \vert ^{p}\varphi)\,dx\,dy:\varphi\in C_{c}^{1} ({R}^{h+1};{R}^{h+1}),\max \vert \varphi \vert \leq1 \}$ *and* $V_{\phi}(E)=\int_{E} \vert x \vert ^{p} \,dx\,dy$ *are called the weighted* *α*-*perimeter and the weighted volume of* *E*, *respectively*. *Finally* $\omega_{h}$ *denotes the Euclidean volume of the unit ball*.

When $p=0$ in Theorem 1.1, we can obtain the quantitative isoperimetric inequalities for the set $E_{\alpha}$ in half-cylinders in Grushin spaces.

## Preliminaries

The Grushin space ${R}^{h+1}=\{(x,y):x\in{R}^{h}, y\in{R}\}$ is a Carnot-Carathéodory space with a system of vector fields

$$X_{i}=\frac{\partial}{\partial x_{i}},\quad i=1,\ldots,h\quad \mbox{and}\quad Y= \vert x \vert ^{\alpha}\frac{\partial}{\partial y}, $$

where $\alpha>0$ is a given real number and $\vert x \vert $ is the standard Euclidean norm of *x*.

The *α*-perimeter of a measurable set $E\subset {R}^{h+1}$ in an open set $A\subset{R}^{h+1}$ is defined as

$$P_{\alpha}(E;A)=\sup \biggl\{ \int_{E} \operatorname{div}_{\alpha}\varphi \,dx\,dy:\varphi \in C_{c}^{1}\bigl(A;{R}^{h+1}\bigr), \Vert \varphi \Vert _{\infty}= \max_{(x,y)\in A} \bigl\vert \varphi(x,y) \bigr\vert \leq1 \biggr\} , $$

where the *α*-divergence of the vector field $\varphi:A\rightarrow{R}^{h+1} $ is given by

$$\operatorname{div}_{\alpha}\varphi=X_{1}\varphi_{1}+ \cdots+X_{h}\varphi _{h}+Y\varphi_{h+1}. $$

If $P_{\alpha}(E;A)<\infty$, by the Riesz representation theorem there exist a positive Radon measure $\mu_{E}$ on *A* and a $\mu_{E}$-measurable function $v_{E}:A\rightarrow{R}^{h+1}$ such that $\vert v_{E} \vert =1$ $\mu_{E}$-a.e. on *A* and the generalized Gauss-Green formula

3 $$ \int_{E}\operatorname{div}_{\alpha}\varphi \,dx\,dy=- \int_{A}\langle\varphi ,v_{E}\rangle \,d \mu_{E} $$

holds for all $\varphi\in C_{c}^{1}(A; {R}^{h+1})$. Here and hereafter, $\langle\cdot,\cdot\rangle$ denotes the standard Euclidean scalar product. The measure $\mu_{E}$ is called *α*-perimeter measure and the function $v_{E}$ is called measure theoretic inner unit *α*-normal of *E*.

Now we endow the Grushin space ${R}^{h+1}$ with density $e^{\phi}$ and define the weighted *α*-perimeter of a measurable set $E\subset{R}^{h+1}$ in an open set $A\subset{R}^{h+1}$ as

4 $$\begin{aligned} P_{\alpha,\phi}(E;A)= {}&\sup \biggl\{ \int_{E} (\operatorname{div}_{\alpha,\phi}\varphi) e^{\phi}\,dx\,dy:\varphi\in C_{c}^{1} \bigl(A;{R}^{h+1}\bigr), \\ & \Vert \varphi \Vert _{\infty}= \max_{(x,y)\in A} \bigl\vert \varphi(x,y) \bigr\vert \leq1 \biggr\} , \end{aligned}$$

where $\operatorname{div}_{\alpha,\phi}\varphi=e^{-\phi}\operatorname {div}_{\alpha}(e^{\phi}\varphi) $ is called the weighted *α*-divergence of *φ*.

By the definition of $\operatorname{div}_{\alpha,\phi }\varphi$, (4) can also be rewritten as

5 $$\begin{aligned} P_{\alpha,\phi}(E;A)= {}&\sup \biggl\{ \int_{E} \operatorname {div}_{\alpha}\bigl(e^{\phi}\varphi\bigr) \,dx\,dy:\varphi\in C_{c}^{1} \bigl(A;{R}^{h+1}\bigr), \\ & \Vert \varphi \Vert _{\infty}= \max_{(x,y)\in A} \bigl\vert \varphi (x,y) \bigr\vert \leq1 \biggr\} . \end{aligned}$$

If $P_{\alpha,\phi}(E;A)<\infty$, then by (3) we have

6 $$ \int_{E}\operatorname{div}_{\alpha,\phi}\varphi \,dV_{\phi}=- \int_{A}\langle\varphi,v_{E}\rangle \,d \mu_{E,\phi}, $$

where $dV_{\phi}=e^{\phi}\,dx\,dy$ is the weighted volume measure and $d\mu_{E,\phi}=e^{\phi}\,d\mu_{E}$ is called the weighted *α*-perimeter measure. For any open set $A\subset{R}^{h+1}$, we have $P_{\alpha,\phi}(E;A)=\mu_{E,\phi}(A)$. When $A={R}^{h+1}$, let $P_{\alpha,\phi}(E)=P_{\alpha,\phi}(E;{R}^{h+1})$.

Let Σ be a hypersurface in the Grushin space ${R}^{h+1}$ with density $e^{\phi}$. Σ can be locally given by the zero set of a function $u\in C^{1}$ such that $\vert \nabla_{\alpha}u \vert \neq0$ on Σ, where $\nabla_{\alpha}u=(X_{1}u,\ldots,X_{h}u,Yu)$ is called the *α*-gradient of *u*. For a set $E= \{(x,y)\in{R}^{h+1}:u(x,y)>0 \}$, the inner unit *α*-normal in equation (6) is given on $\Sigma=\partial E$ by the vector

$$v_{E}=\frac{\nabla_{\alpha}u(x,y)}{ \vert \nabla_{\alpha}u(x,y) \vert }. $$

Then we define the weighted *α*-mean curvature of Σ as

7 $$ H_{\Sigma,\phi}=-\frac{1}{h}\operatorname{div}_{\alpha,\phi}v_{E}=- \frac {1}{h} \bigl(\operatorname{div}_{\alpha}v_{E}+\langle v_{E},\nabla_{\alpha}\phi\rangle \bigr). $$

### Remark 2.1

Noticing that the *α*-mean curvature of Σ is defined by $H_{\Sigma}=-\frac{1}{h}\operatorname{div}_{\alpha}v_{E}$, then from (7) we have

$$H_{\Sigma,\phi}=H_{\Sigma}-\frac{1}{h}\langle v_{E}, \nabla_{\alpha}\phi\rangle. $$

To prove Theorem 1.1, we need the following lemma.

### Lemma 2.1

*Let the Grushin space* ${R}^{h+1}$ *be endowed with density* $e^{\phi}= \vert x \vert ^{p}$. *For any* $0\leq\varepsilon<1$, *let* $C_{\varepsilon}= \{(x,y)\in{R}^{h+1}: \vert x \vert <1, y>y_{\varepsilon}\}$ *be half*-*cylinders*, *where* $y_{\varepsilon}=f(1-\varepsilon)$ *with* $f(r)=\int_{\arcsin r}^{\frac{\pi}{2}}\sin^{\alpha+1}(t)\,dt$. *There exists a continuous function* $u:C_{\varepsilon}\rightarrow{R}$ *with level sets* $\Sigma_{\varepsilon}= \{(x,y)\in C_{\varepsilon}:u(x,y)=s \}$, $s\in{R}$, *such that*

(i) $u\in C^{1}(C_{\varepsilon}\cap E_{\alpha})\cap C^{1}(C_{\varepsilon}\setminus E_{\alpha})$ *and* $\frac{\nabla_{\alpha}u(x,y)}{ \vert \nabla_{\alpha}u(x,y) \vert }$ *is continuously defined on* $C_{\varepsilon}\setminus\{x=0\}$;
(ii) $\bigcup_{s>1}\Sigma_{s}=C_{\varepsilon}\cap E_{\alpha}$ *and* $\bigcup_{s\leq 1}\Sigma_{s}=C_{\varepsilon}\setminus E_{\alpha}$;
(iii) $\Sigma_{s}$ *is a hypersurface of class* $C^{2}$ *with constant weighted* *α*-*mean curvature*, *that is*,
  $$H_{\Sigma_{s},\phi}=\frac{1}{s}\biggl(1+\frac{p}{h}\biggr)\quad\textit{for } s>1 $$
  *and*
  $$H_{\Sigma_{s},\phi}=1+\frac{p}{h}\quad\textit{for } s\leq1; $$
(iv) *for any point* $(x,f( \vert x \vert )-y )\in\Sigma_{s}$ *with* $s>1$, *we have*
  8 $$ 1-\frac{h}{h+p}H_{\Sigma_{s},\phi} \bigl(x,f\bigl( \vert x \vert \bigr)-y \bigr)\geq\frac{1}{5}y^{2}\quad\textit{when } \varepsilon=0 $$
  *and*
  9 $$ 1-\frac{h}{h+p}H_{\Sigma_{s},\phi} \bigl(x,f\bigl( \vert x \vert \bigr)-y \bigr)\geq\frac{\sqrt {\varepsilon}}{(1-\varepsilon)^{\alpha}+\sqrt{\varepsilon}}y \quad\textit{when } 0< \varepsilon< 1. $$

### Proof

The profile function of the set $E_{\alpha}$ is the function $f:[0,1]\rightarrow{R}$,

10 $$ f(r)= \int_{\arcsin r}^{\frac{\pi}{2}}\sin^{\alpha+1}(t)\,dt . $$

Its first and second derivatives are

11 $$ f'(r)=-\frac{r^{\alpha+1}}{\sqrt{1-r^{2}}},\qquad f''(r)= \frac{r^{\alpha}[\alpha r^{2}-(\alpha+1) ]}{(1-r^{2})^{\frac{3}{2}}}. $$

We define the function $g:[0,1]\rightarrow{R}$,

12 $$\begin{aligned} g(r)&=(\alpha+1)f(r)-r f'(r) \\ &=(\alpha+1) \int_{\arcsin r}^{\frac{\pi}{2}}\sin^{\alpha+1}(t)\,dt+ \frac{r^{\alpha+2}}{\sqrt{1-r^{2}}}. \end{aligned}$$

Its derivative is

13 $$ g'(r)=\frac{r^{\alpha+1}}{(1-r^{2})^{\frac{3}{2}}}>0. $$

Now we construct a foliation of $C_{\varepsilon}$. In $C_{\varepsilon}\setminus E_{\alpha}$, the leaves $\Sigma_{s}$ of the foliation are vertical translations of the top part of the boundary $\partial E_{\alpha}$. In $C_{\varepsilon}\cap E_{\alpha}$, the leaves $\Sigma_{s}$ are constructed as follows: the surface $\partial E_{\alpha}$ is dilated by a factor larger than 1 where dilation is defined by $(x,y)\rightarrow(\lambda x,\lambda^{\alpha+1}y)$ $(\forall\lambda>0)$, and then it is translated downwards in such a way that the surface $\{y=y_{\varepsilon}=f(1-\varepsilon)\}$ is also the leaf at last.

We construct a function *u* on the set $C_{\varepsilon}\setminus E_{\alpha}$ as

14 $$ u(x,y)=f\bigl( \vert x \vert \bigr)-y+1,\quad (x,y)\in C_{\varepsilon}\setminus E_{\alpha}. $$

Let $\Sigma_{s}= \{(x,y)\in C_{\varepsilon}\setminus E_{\alpha}:u(x,y)=s \} $. Then we have $s\leq1$ and $\Sigma_{1}=\partial E_{\alpha}$. From (14), we know $u\in C^{1}(C_{\varepsilon}\setminus E_{\alpha})$ and $\bigcup_{s\leq 1}\Sigma_{s}=C_{\varepsilon}\setminus E_{\alpha}$.

In the following we will define the function *u* on the set $D_{\varepsilon}=C_{\varepsilon}\cap E_{\alpha}$. Setting $r= \vert x \vert $ and $r_{\varepsilon}=1-\varepsilon$, we let $F_{\varepsilon}:D_{\varepsilon}\times(1,\infty)\rightarrow{R}$ be a function

15 $$ F_{\varepsilon}(x,y,s)=s^{\alpha+1} \biggl[f\biggl( \frac{r}{s}\biggr)-f\biggl(\frac {r_{\varepsilon}}{s}\biggr) \biggr]+y_{\varepsilon}-y. $$

For any $(x,y)\in D_{\varepsilon}$ we have

$$\lim_{s\rightarrow1^{+}}F_{\varepsilon}(x,y,s)=f(r)-y>0,\qquad \lim _{s\rightarrow\infty}F_{\varepsilon}(x,y,s)=y_{\varepsilon}-y< 0. $$

On the other hand, using (12) and (13) we have

16 $$ \partial_{s} F_{\varepsilon}=s^{\alpha}\biggl[g\biggl(\frac{r}{s}\biggr)-g\biggl(\frac{r_{\varepsilon}}{s}\biggr) \biggr]< 0. $$

So there exists a unique $s>1$ such that $F_{\varepsilon}(x,y,s)=0$ for any $(x,y)\in D_{\varepsilon}$. Furthermore we can define a function $u:D_{\varepsilon}\rightarrow{R},s=u(x,y)$ determined by the equation $F_{\varepsilon}(x,y,s)=0$. Obviously we have $u\in C^{1}(C_{\varepsilon}\cap E_{\alpha})$ and $C_{\varepsilon}\cap E_{\alpha}=\bigcup_{s>1}\Sigma_{s}$, where $\Sigma_{s}= \{(x,y)\in C_{\varepsilon}\cap E_{\alpha}:s=u(x,y) \mbox{ is determined by }F_{\varepsilon}(x,y,s)=0 \}$.

By (15), we find

17 $$ \partial_{x_{i}}F_{\varepsilon}(x,y,s)= \frac{s^{\alpha}x_{i}}{r}f'\biggl(\frac{r}{s}\biggr),\quad i=1,\ldots,h;\qquad \partial_{y}F_{\varepsilon}(x,y,s)=-1. $$

Using (11), (16) and (17), we obtain

18 $$ \partial_{x_{i}}u(x,y)= \frac{x_{i}r^{\alpha}}{s^{\alpha}\sqrt{s^{2}-r^{2}} [g(\frac{r}{s})-g(\frac {r_{\varepsilon}}{s}) ]},\qquad \partial_{y}u(x,y)= \frac{1}{s^{\alpha}[g(\frac{r}{s})-g(\frac{r_{\varepsilon}}{s}) ]}. $$

Then we have

$$X_{i}u=\partial_{x_{i}}u=\frac{x_{i}r^{\alpha}}{s^{\alpha}\sqrt{s^{2}-r^{2}} [g(\frac{r}{s})-g(\frac{r_{\varepsilon}}{s}) ]},\qquad Yu= \vert x \vert ^{\alpha}\partial_{y}u=\frac{r^{\alpha}}{s^{\alpha}[g(\frac {r}{s})-g(\frac{r_{\varepsilon}}{s})]}, $$

and the square length of the *α*-gradient of *u* on $D_{\varepsilon}$ is

$$\vert \nabla_{\alpha}u \vert ^{2}=\sum _{i=1}^{h}(X_{i}u)^{2}+(Yu)^{2}= \frac{r^{2\alpha}}{ s^{2\alpha-2}(s^{2}-r^{2}) [g(\frac{r}{s})-g(\frac{r_{\varepsilon}}{s}) ]^{2}}. $$

Note that $\vert \nabla_{\alpha}u \vert =0$ if and only if $x=0$. So for any $(x,y)\in D_{\varepsilon}$ with $x\neq0$, we have

19 $$ \frac{X_{i}u}{ \vert \nabla_{\alpha}u \vert }=-\frac{x_{i}}{s}, \quad i=1,\ldots,h;\qquad \frac{Yu}{ \vert \nabla_{\alpha}u \vert }=-\frac{\sqrt{s^{2}-r^{2}}}{s}. $$

If $(x,y)\in D_{\varepsilon}$ tends to $(\overline{x},\overline{y})\in \partial E_{\alpha}$ with $\overline{x}\neq0$ and $\overline{y}>0$, then $s=u(x,y)$ converges to 1. From (19), we have

$$\lim_{(x,y)\rightarrow(\overline{x},\overline{y})}\frac{\nabla_{\alpha}u(x,y)}{ \vert \nabla_{\alpha}u(x,y) \vert } = \bigl(-\overline{x}_{1}, \ldots,-\overline{x}_{h},\sqrt{1- \vert \overline {x} \vert ^{2}} \bigr)=\frac{\nabla_{\alpha}u(\overline{x},\overline{y})}{ \vert \nabla_{\alpha}u(\overline{x},\overline{y}) \vert }, $$

where the right hand side is computed by the definition (14) of *u*. The above equality shows that $\frac{\nabla_{\alpha}u}{ \vert \nabla_{\alpha}u \vert }$ is continuous on $C_{\varepsilon}\setminus\{x=0\}$.

In the case of $e^{\phi}= \vert x \vert ^{p}$, we get $\phi=p\ln \vert x \vert $ and $\nabla_{\alpha}\phi= (\frac{p}{ \vert x \vert ^{2}}x_{1},\ldots,\frac {p}{ \vert x \vert ^{2}}x_{h},0 )$ for $x\neq0$. From (14), we know that the inner unit *α*-normal of $\Sigma_{s}$ with $s\leq1$ is

$$v_{\Sigma_{s}}= \bigl(-x_{1},\ldots,-x_{h},-\sqrt{1- \vert x \vert ^{2}} \bigr). $$

So the weighted *α*-mean curvature $H_{\Sigma_{s},\phi}$ of $\Sigma_{s}$ with $s\leq1$ is given by

$$H_{\Sigma_{s},\phi}=\frac{1}{h} \bigl(-\operatorname{div}_{\alpha}v_{E_{\alpha}}-\langle v_{E_{\alpha}},\nabla_{\alpha}\phi\rangle \bigr)=1+\frac{p}{h}. $$

From (19) we know that the inner unit *α*-normal of $\Sigma_{s}$ with $s>1$ is

$$v_{\Sigma_{s}}= \biggl(-\frac{x_{1}}{s},\ldots,-\frac{x_{h}}{s},- \frac{\sqrt {s- \vert x \vert ^{2}}}{s} \biggr). $$

So the weighted *α*-mean curvature $H_{\Sigma_{s},\phi}$ of $\Sigma_{s}$ with $s>1$ is given by

$$H_{\Sigma_{s},\phi}=\frac{1}{s}\biggl(1+\frac{p}{h}\biggr). $$

Fixing a point *x* with $\vert x \vert <1-\varepsilon$ and for $0\leq y< f( \vert x \vert )-y_{\varepsilon}$, we define the function

20 $$ h_{x}(y)=u \bigl(x,f\bigl( \vert x \vert \bigr)-y \bigr)=s=\biggl(1+\frac{p}{h}\biggr)\frac{1}{H_{\Sigma_{s},\phi}}, $$

where $s\geq1$ is uniquely determined by $(x,f( \vert x \vert )-y)\in \Sigma_{s}$. Then the function $y\rightarrow h_{x}(y)$ is increasing and $h_{x}(0)=1$.

From (18) and (20), we know

$$h_{x}'(y)=-\partial_{y}u \bigl(x,f\bigl( \vert x \vert \bigr)-y \bigr)=\frac{1}{ (h_{x}(y) )^{\alpha}[g(\frac{r_{\varepsilon}}{h_{x}(y)})-g(\frac{r}{h_{x}(y)}) ]}, $$

for all $0\leq y< f( \vert x \vert )-y_{\varepsilon}$.

By (13), *g* is strictly increasing. So $h_{x}(y)$ satisfies

21 $$ h_{x}'(y)\geq \frac{1}{h_{x}^{\alpha}(y) [g(\frac{r_{\varepsilon}}{h_{x}(y)})-g(0) ]}. $$

On the other hand, for any $s>1$ we have

22 $$\begin{aligned} s^{\alpha}\biggl[g\biggl(\frac{r_{\varepsilon}}{s}\biggr)-g(0) \biggr]&=s^{\alpha}\int _{0}^{\frac{r_{\varepsilon}}{s}}g'(r)\,dr \\ &=s^{\alpha}\int_{0}^{\frac{r_{\varepsilon}}{s}}\frac{r^{\alpha +1}}{(1-r^{2})^{\frac{2}{3}}}\,dr \\ &\leq r_{\varepsilon}^{\alpha}\int_{0}^{\frac{r_{\varepsilon}}{s}}\frac {r}{(1-r^{2})^{\frac{2}{3}}}\,dr \\ &=r_{\varepsilon}^{\alpha}\biggl[\biggl(1-\biggl(\frac{r_{\varepsilon}}{s} \biggr)^{2}\biggr)^{-\frac {1}{2}}-1 \biggr] \\ &\leq r_{\varepsilon}^{\alpha}\frac{s}{\sqrt{s^{2}-r_{\varepsilon}^{2}}} \\ &\leq r_{\varepsilon}^{\alpha}\frac{1}{\sqrt{s-r_{\varepsilon}}}. \end{aligned}$$

When $\varepsilon=0$, we have $r_{\varepsilon}=1$. So (22) turns into

$$s^{\alpha}\biggl[g\biggl(\frac{r_{\varepsilon}}{s}\biggr)-g(0) \biggr]\leq \frac{1}{\sqrt{s-1}}. $$

By (21), we get

23 $$ h_{x}'(y)\geq\sqrt{h_{x}(y)-1},\quad y \geq0. $$

Integrating (23) with $h_{x}(0)=1$, we get

$$h_{x}(y)\geq1+\frac{1}{4}y^{2}. $$

Thus we obtain

$$1-\frac{h}{h+p}H_{\Sigma_{s},\phi} \bigl(x,f\bigl( \vert x \vert \bigr)-y \bigr)=1-\frac {1}{h_{x}(y)}\geq\frac{y^{2}}{4+y^{2}}. $$

Noticing

$$y < f\bigl( \vert x \vert \bigr) \leq f(0)= \int_{0}^{\frac{\pi}{2}}\sin^{\alpha+1}(t)\,dt\leq \int_{0}^{\frac{\pi}{2}}\sin(t)\,dt =1, $$

we have

24 $$ 1-\frac{h}{h+p}H_{\Sigma_{s},\phi} \bigl(x,f\bigl( \vert x \vert \bigr)-y \bigr)\geq\frac{1}{5}y^{2}. $$

When $0<\varepsilon<1$, (22) turns into

$$s^{\alpha}\biggl[g\biggl(\frac{r_{\varepsilon}}{s}\biggr)-g(0) \biggr]\leq \frac {(1-\varepsilon)^{\alpha}}{\sqrt{s-1+\varepsilon}}. $$

So by (21), we have

25 $$ h_{x}'(y)\geq\frac{\sqrt{\varepsilon}}{(1-\varepsilon)^{\alpha}},\quad y\geq0. $$

Integrating (25) with $h_{x}(0)=1$, we have

$$h_{x}(y)\geq1+\frac{\sqrt{\varepsilon}}{(1-\varepsilon)^{\alpha}}y. $$

Noticing $y< f( \vert x \vert )-y_{\varepsilon}\leq1$, so we have

26 $$\begin{aligned} 1-\frac{h}{h+p}H_{\Sigma_{s},\phi} \bigl(x,f\bigl( \vert x \vert \bigr)-y \bigr)&=1-\frac {1}{h_{x}(y)} \\ &\geq 1-\frac{1}{1+\frac{\sqrt{\varepsilon}}{(1-\varepsilon)^{\alpha}}y} \\ &\geq \frac{\sqrt{\varepsilon}}{(1-\varepsilon)^{\alpha}+\sqrt{\varepsilon}}y. \end{aligned}$$

 □

## Proof of Theorem 1.1

Let $u:C_{\varepsilon}\rightarrow{R}$ be the function given by Lemma 2.1 and let $\Sigma_{s}= \{(x,y)\in C_{\varepsilon}:u(x,y)=s \}$ be leaves of the foliation, $s\in{R}$. We define the vector field $X:C_{\varepsilon}\setminus\{x=0\}\rightarrow{R}^{h+1}$ by

$$X=-\frac{\bigtriangledown_{\alpha}u}{ \vert \bigtriangledown_{\alpha}u \vert }. $$

Then *X* satisfies the following properties:

(i) $\vert X \vert =1$;
(ii) for $(x,y)\in\partial E_{\alpha}\cap C_{\varepsilon}$, we have $X(x,y)=-v_{E_{\alpha}}(x,y)$ where $v_{E_{\alpha}}(x,y)$ is the unit inner *α*-normal to $\partial E_{\alpha}$;
(iii) for any point $(x,y)\in\Sigma_{s}$ with $s\leq1$, we have
  27 $$ \operatorname{div}_{\alpha,\phi} X(x,y)=h+p. $$

For any point $(x,y)\in\Sigma_{s}$ with $s>1$, we have

28 $$ \operatorname{div}_{\alpha,\phi} X(x,y)=\frac{1}{s}(h+p)< h+p. $$

Let $F\subset{R}^{h+1}$ be a set with finite weighted *α*-perimeter such that $V_{\phi}(F)=V_{\phi}(E_{\alpha})$ and $F\triangle E_{\alpha}\subset\subset C_{\varepsilon}$. By Theorem 2.2.2 in [27], without loss of generality we can assume that the boundary *∂F* of *F* is $C^{\infty}$.

For $\delta>0$, let $E_{\alpha}^{\delta}= \{(x,y)\in E_{\alpha}: \vert x \vert >\delta \}$. By (28) and (6), we have

$$\begin{aligned} V_{\phi}\bigl(E_{\alpha}^{\delta}\setminus F\bigr)&= \int_{E_{\alpha}^{\delta}\setminus F} \vert x \vert ^{p} \,dx\,dy \\ &\geq \int_{E_{\alpha}^{\delta}\setminus F}\frac{\operatorname{div}_{\alpha ,\phi} X}{h+p} \vert x \vert ^{p} \,dx\,dy \\ &=\frac{1}{h+p} \biggl\{ \int_{\partial F\cap E_{\alpha}^{\delta}}\langle X,v_{F}\rangle \,d\mu_{F,\phi}- \int_{\partial E_{\alpha}^{\delta}\cap F}\langle X,v_{E_{\alpha}^{\delta}}\rangle \,d\mu_{{E_{\alpha}^{\delta}},\phi} \biggr\} . \end{aligned}$$

Letting $\delta\rightarrow0^{+}$ and using the Cauchy-Schwarz inequality, we obtain

29 $$\begin{aligned} V_{\phi}(E_{\alpha}\setminus F)&= \int_{E_{\alpha}\setminus F} \vert x \vert ^{p} \,dx\,dy \\ &\geq \int_{E_{\alpha}\setminus F}\frac{\operatorname{div}_{\alpha,\phi} X}{h+p} \vert x \vert ^{p} \,dx\,dy \\ &=\frac{1}{h+p} \biggl\{ \int_{\partial F\cap E_{\alpha}}\langle X,v_{F}\rangle \,d\mu_{F,\phi}- \int_{\partial E_{\alpha}\setminus F}\langle X,v_{E_{\alpha}}\rangle \,d\mu_{E_{\alpha},\phi} \biggr\} \\ &\geq \frac{1}{h+p} \biggl\{ \int_{\partial E_{\alpha}\setminus F}\,d\mu_{E_{\alpha},\phi}- \int_{\partial F\cap E_{\alpha}}\,d\mu_{F,\phi} \biggr\} \\ &=\frac{1}{h+p} \bigl\{ P_{\alpha,\phi}(E_{\alpha};C_{\varepsilon}\setminus F)-P_{\alpha,\phi}(F;E_{\alpha}) \bigr\} . \end{aligned}$$

By a similar computation, we also have

30 $$\begin{aligned} &V_{\phi}(F\setminus E_{\alpha}) \\ &\quad = \int_{F\setminus E_{\alpha}} \vert x \vert ^{p} \,dx\,dy \\ &\quad= \int_{F\setminus E_{\alpha}}\frac{\operatorname{div}_{\alpha,\phi} X}{h+p} \vert x \vert ^{p} \,dx\,dy \\ &\quad=\frac{1}{h+p} \biggl\{ - \int_{\partial F\setminus E_{\alpha}}\langle X,v_{F}\rangle \,d\mu_{F,\phi}+ \int_{\partial E_{\alpha}\cap F}\langle X,v_{E_{\alpha}}\rangle \,d\mu_{E_{\alpha},\phi} \biggr\} \\ &\quad\leq\frac{1}{h+p} \biggl\{ \int_{\partial F\setminus E_{\alpha}}\,d\mu_{F,\phi}- \int_{\partial E_{\alpha}\cap F}\,d\mu_{E_{\alpha},\phi } \biggr\} \\ &\quad=\frac{1}{h+p} \bigl\{ P_{\alpha,\phi}(F;C_{\varepsilon}\setminus E_{\alpha})-P_{\alpha,\phi}(E_{\alpha};F) \bigr\} . \end{aligned}$$

On the other hand, we have

31 $$\begin{aligned} \int_{E_{\alpha}\setminus F}\frac{\operatorname{div}_{\alpha,\phi}X}{h+p} \vert x \vert ^{p} \,dx\,dy &= \int_{E_{\alpha}\setminus F} \biggl[1+\biggl(\frac{\operatorname{div}_{\alpha ,\phi} X}{h+p}-1\biggr) \biggr] \vert x \vert ^{p} \,dx\,dy \\ &= V_{\phi}(E_{\alpha}\setminus F)- \int_{E_{\alpha}\setminus F}\biggl(1-\frac{\operatorname{div}_{\alpha,\phi} X}{h+p}\biggr) \vert x \vert ^{p} \,dx\,dy. \end{aligned}$$

From (29), (30) and (31), we obtain

$$\begin{aligned} &\frac{1}{h+p} \bigl\{ P_{\alpha,\phi}(E_{\alpha};C_{\varepsilon}\setminus F )-P_{\alpha,\phi}(F;E_{\alpha}) \bigr\} \\ &\quad\leq \int_{E_{\alpha}\setminus F}\frac{\operatorname{div}_{\alpha,\phi} X}{h+p} \vert x \vert ^{p} \,dx\,dy \\ &\quad =V_{\phi}(E_{\alpha}\setminus F)- \int_{E_{\alpha}\setminus F}\biggl(1-\frac{\operatorname{div}_{\alpha,\phi} X}{h+p}\biggr) \vert x \vert ^{p} \,dx\,dy \\ &\quad =V_{\phi}(F\setminus E_{\alpha})- \int_{E_{\alpha}\setminus F}\biggl(1-\frac{\operatorname{div}_{\alpha,\phi} X}{h+p}\biggr) \vert x \vert ^{p} \,dx\,dy \\ &\quad \leq \frac{1}{h+p} \bigl\{ P_{\alpha,\phi}(F;C_{\varepsilon}\setminus E_{\alpha})-P_{\alpha,\phi}(E_{\alpha};F) \bigr\} - \int_{E_{\alpha}\setminus F}\biggl(1-\frac{\operatorname{div}_{\alpha,\phi} X}{h+p}\biggr) \vert x \vert ^{p} \,dx\,dy. \end{aligned}$$

It is equivalent to

32 $$ P_{\alpha,\phi}(F)-P_{\alpha,\phi}(E_{\alpha})\geq (h+p) \int_{E_{\alpha}\setminus F}\biggl(1-\frac{\operatorname{div}_{\alpha,\phi} X}{h+p}\biggr) \vert x \vert ^{p}\,dx\,dy. $$

For any *x* with $\vert x \vert <1-\varepsilon$, we define the vertical sections $E_{\alpha}^{x}= \{y:(x,y)\in E_{\alpha}\}$ and $F^{x}= \{y:(x,y)\in F \}$. By the Fubini theorem, we have

$$\begin{aligned} &\int_{E_{\alpha}\setminus F}\biggl(1-\frac{\operatorname{div}_{\alpha,\phi} X}{h+p}\biggr) \vert x \vert ^{p}\,dx\,dy\\ &\quad= \int_{ \{ \vert x \vert < 1-\varepsilon \}} \int_{E_{\alpha}^{x}\setminus F^{x}}\biggl(1-\frac{\operatorname{div}_{\alpha,\phi} X}{h+p}\biggr) \vert x \vert ^{p}\,dy\,dx. \end{aligned}$$

Letting $m(x)={\mathcal{L}}^{1}(E_{\alpha}^{x}\setminus F^{x})$, where $\mathcal{ L}^{1}$ denotes 1-dimensional Lebesgue measure, then we obtain

33 $$\begin{aligned} \int_{E_{\alpha}\setminus F}\biggl(1-\frac{\operatorname{div}_{\alpha,\phi} X}{h+p}\biggr) \vert x \vert ^{p}\,dx\,dy &= \int_{ \{ \vert x \vert < 1-\varepsilon \}} \int _{f( \vert x \vert )-m(x)}^{f( \vert x \vert )}\biggl(1-\frac{\operatorname{div}_{\alpha,\phi} X}{h+p}\biggr) \vert x \vert ^{p}\,dy\,dx \\ &= \int_{ \{ \vert x \vert < 1-\varepsilon \}} \int_{0}^{m(x)} \biggl(1-\frac {1}{h_{x}(y)} \biggr) \vert x \vert ^{p}\,dy\,dx, \end{aligned}$$

where $h_{x}(y)=u (x,f( \vert x \vert )-y )$ is the function introduced in (20).

So from (32) and (33) we have

34 $$ P_{\alpha,\phi}(F)-P_{\alpha,\phi}(E_{\alpha})\geq (h+p) \int_{ \{ \vert x \vert < 1-\varepsilon \}} \int_{0}^{m(x)} \biggl(1-\frac {1}{h_{x}(y)} \biggr) \vert x \vert ^{p}\,dy\,dx. $$

When $\varepsilon=0$, by (8) in Lemma 2.1 and the Hölder inequality, (34) turns into

35 $$\begin{aligned} P_{\alpha,\phi}(F)-P_{\alpha,\phi}(E_{\alpha})&\geq (h+p) \int_{ \{ \vert x \vert < 1 \}} \int_{0}^{m(x)}\frac{1}{5}y^{2}\,dy \vert x \vert ^{p}\,dx \\ &\geq \frac{h+p}{15} \int_{ \{ \vert x \vert < 1 \}} \bigl(m(x) \bigr)^{3} \vert x \vert ^{3p}\,dx \\ &\geq \frac{h}{15\omega_{h}^{2}} \biggl( \int_{ \{ \vert x \vert < 1 \}}m(x) \vert x \vert ^{p} \,dx \biggr)^{3} \\ &= \frac{h+p}{120\omega_{h}^{2}}V_{\phi}(E_{\alpha}\triangle F)^{3}. \end{aligned}$$

When $0<\varepsilon<1$, by (9) in Lemma 2.1, and the Hölder inequality, (34) turns into

36 $$\begin{aligned} P_{\alpha,\phi}(F)-P_{\alpha,\phi}(E_{\alpha})&\geq (h+p) \int_{ \{ \vert x \vert < 1-\varepsilon \}} \int_{0}^{m(x)}\frac{\sqrt {\varepsilon}}{(1-\varepsilon)^{\alpha}+ \sqrt{\varepsilon}}y\,dy \vert x \vert ^{p}\,dx \\ &\geq \frac{\sqrt{\varepsilon}(h+p)}{2 [(1-\varepsilon)^{\alpha}+\sqrt {\varepsilon} ]} \int_{ \{ \vert x \vert < 1-\varepsilon \}} \bigl(m(x) \bigr)^{2} \vert x \vert ^{2p}\,dx \\ &\geq \frac{\sqrt{\varepsilon}(h+p)}{2 [(1-\varepsilon)^{\alpha +h}+(1-\varepsilon)^{h}\sqrt{\varepsilon} ]\omega_{h}} \biggl( \int_{ \{ \vert x \vert < 1-\varepsilon \}}m(x) \vert x \vert ^{p}\,dx \biggr)^{2} \\ &=\frac{\sqrt{\varepsilon}(h+p)}{8 [(1-\varepsilon)^{\alpha +h}+(1-\varepsilon)^{h}\sqrt{\varepsilon} ]\omega_{h}}V_{\phi}(E_{\alpha}\triangle F)^{2}. \end{aligned}$$
